# Complement Factor H in cSCC: Evidence of a Link Between Sun Exposure and Immunosuppression in Skin Cancer Progression

**DOI:** 10.3389/fonc.2022.819580

**Published:** 2022-02-10

**Authors:** Ellise M. Johnson, Chandana K. Uppalapati, Agnes S. Pascual, Sarah I. Estrada, Richard L. Averitte, Kathryn J. Leyva, Elizabeth E. Hull

**Affiliations:** ^1^ Biomedical Sciences Program, College of Graduate Studies, Midwestern University, Glendale, AZ, United States; ^2^ Department of Microbiology and Immunology, College of Graduate Studies, Midwestern University, Glendale, AZ, United States; ^3^ Affiliated Dermatology & Affiliated Laboratories, Scottsdale, AZ, United States

**Keywords:** cutaneous squamous cell carcinoma (cSCC), Complement Factor H, immunomodulation, FOXP3, interferon gamma (IFNγ), sun exposure, complement cascade, immunoevasion

## Abstract

Cutaneous squamous cell carcinoma (cSCC) is a common form of skin cancer with an estimated 750,000 cases diagnosed annually in the United States. Most cases are successfully treated with a simple excision procedure, but ~5% of cases metastasize and have a 5-year survival rate of 25-45%. Thus, identification of biomarkers correlated to cSCC progression may be useful in the early identification of high-risk cSCC and in the development of new therapeutic strategies. This work investigates the role of complement factor H (CFH) in the development of cSCC. CFH is a regulatory component of the complement cascade which affects cell mediated immune responses and increases in complement proteins are associated with poor outcomes in multiple cancer types. We provide evidence that sun exposure may increase levels of CFH, suggesting an immunomodulatory role for CFH early in the development of cSCC. We then document increased levels of CFH in cSCC samples, compared to adjacent normal tissue (ANT) routinely excised in a dermatology clinic which, in paired samples, received the same level of sun exposure. We also provide evidence that levels of CFH are even greater in more advanced cases of cSCC. To provide a potential link between CFH and immune modulation, we assessed immune system function by measuring interferon gamma (IFN-γ) and FOXP3 in patient samples. IFN-γ levels were unchanged in cSCC relative to ANT which is consistent with an ineffective cell-mediated immune response. FOXP3 was used to assess prevalence of regulatory T cells within the tissues, indicating either a derailed or inhibitory immune response. Our data suggest that FOXP3 levels are higher in cSCC than in ANT. Our current working model is that increased CFH downstream of sun exposure is an early event in the development of cSCC as it interferes with proper immune surveillance and decreases the effectiveness of the immune response, and creates a more immunosuppressive environment, thus promoting cSCC progression.

## Introduction

Cutaneous squamous cell carcinoma (cSCC) is typically treated by tumor excision with a success rate of >95%. As a minority of cSCC are known to metastasize and cause clinically serious disease, research on cSCC is sparse and therapies for the ~5% of cases that do metastasize are limited, resulting in a 5-year survival rate of only 25-45% (1, 2). However, as the incidence of cSCC is increasing (3), an understanding of the factors that may increase the ability of these tumors to metastasize is of particular importance.

Evidence is accumulating that the tumor microenvironment is a key factor in the progression of all tumor types. The immunomodulatory nature of the tumor microenvironment has been shown to be particularly relevant due to discovery of the clinical efficacy of treatments targeting immune checkpoints. In this work, we focus on the potential role of two complement regulatory proteins, complement factor H (CFH) and complement factor I (CFI), in the immune response to tumors.

As regulatory proteins, CFH and CFI modulate the complement cascade at multiple points, but their most impactful effect is through reducing levels of several potent anaphylatoxins (including C3a and C5a). Local anaphylatoxin production increases recruitment of both innate and adaptive immune cells to the tumor. In addition, recently characterized as immune checkpoints (4), C3aR and C5aR signaling modulates the T cell response by promoting T cell survival and favoring differentiation of pro-inflammatory Th1 effector cells over immunosuppressive FOXP3+ regulatory T cells. Thus, the complement system is an integral part of a coordinated immune (5) response to tumors. As CFH and CFI are known to decrease levels of C3a and C5a, these two complement regulatory proteins function to dampen cell-mediated immune responses in inflammation and, although many questions still remain, have been shown to decrease immune responses by non-canonical mechanisms (5, 6).

Complement regulatory proteins may also play a direct role in promoting cSCC development. Keratinocytes have been shown to synthesize both CFH and CFI, as well as other complement proteins (7–10). Suggestive of a functional role, these regulatory proteins were shown to increase migration and proliferation when added to cSCC cell cultures and CFI appears to be related to tumor growth *in vivo* (7, 8). Interestingly, synthesis of CFH and CFI by human keratinocytes is upregulated by the pro-inflammatory cytokine interferon gamma (IFN-γ) (5–7). This suggests that cSCC may have the ability to upregulate complement regulatory proteins to actively derail the immune response to tumors once an immune response to a tumor is established. Thus, elevated complement regulatory expression may directly promote tumor survival and metastasis in addition to derailing the immune response to tumors.

To underscore the clinical importance of these regulatory components, analysis of the TCGA dataset reveals that CFH and CFI expression are unfavorable prognostic markers in renal and urothelial cancers respectively (11), and several studies have identified CFH as a cancer biomarker (12–14). Furthermore, clinical therapies using anaphylatoxin receptor antagonists and anti-CFH antibodies are being investigated (4, 15). In addition, recent data suggests that the role of complement in tumorigenesis is unexpectedly complex. Several complement components increase ERK 1/2 and it is interesting that these components both promote and inhibit formation of membrane attack complex (MAC) (7, 8, 16, 17). In addition, CFH has recently been shown to have intracellular activities and to promote tumor progression independently of the canonical extracellular role of complement (6, 18–20).

In this work, we seek to extend the understanding of the role of these complement regulatory proteins in the development of cSCC. First, we ask if sun exposure alters CFH and CFI expression using existing datasets. Second, focusing on cSCC tissue samples removed from patients routinely seen in a dermatology clinic, we ask if a difference in CFH levels can be detected in cSCC tissue samples compared to adjacent normal tissue; these paired tissue samples received the same level of sun exposure. Third, we ask if there is a shift in the cell-mediated immune response between cSCC and adjacent normal tissues by assaying IFN-γ and FOXP3 levels.

## Methods

### Patient Consent and Tissue Collection

All experimentation on human tissue samples was approved by Western IRB (WIRB Protocol #20142461) to Affiliated Laboratories BioRepository (ALBR). Additionally, the Midwestern Institutional Review Board approved the use of these clinic-based biorepository samples at Midwestern University (AZ#807). The single criterion for the collection of tissues for these procedures is a biopsy-proven diagnosis of cSCC. The initial diagnosis and classification of cSCC type was completed at the clinic as part of routine patient care prior to transfer of the sample to the research laboratory. No exclusion criteria were outlined in the original IRB protocol but samples from patients with a known blood-borne communicable disease were not used. All tissue specimens were obtained from patients who consented to donate excised tissue removed during Mohs surgery. For viable tissue used in explant cultures, cSCC tissue from the center of the apical side of the tumor was removed before processing the sample for histology. If needed for wound closure, the surgeon removed adjacent normal tissue (ANT) and these were matched with the tumor sample for paired analysis.

### Explant Culture and Immunofluorescence of cSCC and ANT

Tissues were processed for culture and immunofluorescence as described in Belden et al. (21). Briefly, post-Mohs tissue was rinsed briefly in 70% ethanol to sterilize, covered with media, minced with a razor blade, and placed into 35 mm culture dishes. 20 µl of fetal bovine serum (FSB) was placed in each culture dish to cover tissue slices and left to dry in the culture hood for 20 minutes. 1 ml of culture media (1:1 mixture of DMEM : Ham’s F-12 supplemented with 10% FBS, 25 mM Hepes, and 100 IU/ml of penicillin and 100 µg/ml streptomycin) was then added to each dish/tissue slice and incubated at 37°C in a humidified CO_2_ incubator. When approximately 80% confluent, cultures of mixed cultures were passaged onto glass coverslips for immunofluorescence.

For immunofluorescence, explant cells were grown on eight-well chamber slides, washed with 1x PBS, fixed for 15 minutes with 4% paraformaldehyde in 1x PBS, rinsed with 1x PBS, and incubated in 0.05% Triton X-100 in 1x PBS for 5 minutes to permeabilize the cells, and followed by blocking with 1% BSA in 1x PBS for one hour. Blocking reagent was aspirated and cells were rinsed with 1x PBS and incubated overnight with 1:200 mouse anti-CFH (Abnova, OX-24) at room temperature. Primary antibody was omitted as a negative control. After washing with 1x PBS, cells were incubated with Alexa Fluor 488 goat anti-mouse and Alexa Fluor 568 phalloidin (1:500) for one hour at room temperature, washed with 1x PBS, mounted in fluoromount with DAPI (Electron Microscopy Sciences) and imaged with a Zeiss Apotome microscope.

### Immunoblotting

Total protein from patient derived frozen tissue samples (Affiliated Dermatology Laboratory) were isolated using RIPA buffer (50 mM Tris HCl, pH 8.0; 150 mM NaCl; 1% NP-40; 0.5% sodium deoxycholate; 0.1% SDS; HALT (Protease and Phosphatase Inhibitor), DNaseI and DTT following an established protocol (21). Forty micrograms of total protein from each sample were resolved on either a 10% (FOXP3) or 4-20% (all other proteins) Mini-PROTEAN^®^ TGX™ Precast Protein gel (Bio-Rad), transferred to a low fluorescent PVDF membrane, and blocked using 5% NFDM (non-fat dry milk, 1X TBS, 0.1% Tween 20) for one hour at room temperature. Primary antibodies in 1% NFDM used were 1:200 rabbit monoclonal histone H3 antibody (D1H2) (Cell Signaling Technology), 1:1000 rabbit anti-GAPDH (Cell Signaling Technology), 1:200 mouse monoclonal CFH (OX-24) (Abnova, OX-24), 1:200 mouse monoclonal IFN-γ (Santa Cruz Biotechnology), and 1:200 mouse monoclonal antibody FOXP3 (F9) (Santa Cruz Biotechnology). 1:10,000 AlexaFluor^®^ 790 (Abcam) or 1:5,000 HRP-conjugated (Santa Cruz Biotechnology) were used as secondary antibodies. All blots were performed in triplicate and relative protein expression was measured using either an Odyssey^®^ CLx (LI-COR Biotechnology) or ChemiDoc XRS (BioRad) imaging system. Band intensities were normalized to either GAPDH or H3 using Image J software (NIH).

### Immunohistochemistry

Slides of formalin-fixed, paraffin-embedded (FFPE) cSCC and ANT tissue sections were either purchased (US Biomax & Biochain) or obtained from ALBR. A standard immunohistochemistry protocol was performed by baking the sections at 60°C for 60 minutes, de-paraffinizing by placing in xylene followed by reducing concentrations of ethanol (100% to 70%). De-paraffined sections were permeabilized using 0.25% Trypsin with no EDTA. For heat induced epitope retrieval, FFPE tissue sections were incubated in either citrate buffer (CFH) or basic buffer (FOXP3) at 95°C for 25 minutes, followed by blocking and overnight incubations at 37°C, and 4°C with primary antibodies, mouse anti-CFH (OX-24) (Novus Biological) and rabbit monoclonal anti-FOXP3 (Cell Marque). CFH slides were incubated with AP-conjugated secondary and permanent red stain. FOXP3 slides were incubated with an HRP-conjugated secondary and DAB stain (for array slides) or an AP-conjugated secondary antibody and permanent red stain (for ALBR slides). Mayer’s Hematoxylin was used as a counterstain (nuclei) followed by mounting with Flourmount G mounting medium. Slides were imaged using bright field Olympus (DP73 camera) microscope at 40X, 100X and 400X magnifications. An isotype control was performed for each tissue type and against each antibody species. Semi-quantitative analysis of IHC images was performed based on colorimetric intensity over a specified area of tissue sections using a 0-3+ scale, with 0 indicating no staining, 1+ indicating <10% staining, 2+ indicating 10-50% staining, and 3+ indicating >50% staining, and verified using ImageJ (NIH) (22).

### Statistical Analysis

Analysis of GTEx (Genotype-Tissue Expression) data was performed using non-parametric tests in GraphPad Prism v9. A Mann-Whitney two-tailed T-test was used to analyse unpaired data and a two-tailed Wilcoxon matched-pair signed-rank test was used to analyse paired data. Statistical significance did not vary with or without removal of outliers using the ROUT method of identifying outliers. Correlation of paired data was performed using Spearman’s rank-order correlation coefficient.

Band intensities from immunoblot data were normalized to histone H3 or GAPDH. A ROUT test with an alpha value of 0.05 was used to identify potential outliers within each dataset. D’Agostino and Shapiro-Wilk tests followed by a T-test (paired or unpaired depending on the comparison) were used for determining the significance of differences for normally distributed independent variables. D’Agostino and Shapiro-Wilk tests followed by a Mann–Whitney U-test was used for determining the significance of differences between two non-normally distributed independent variables. A *post-hoc* power analysis was applied to data that did not show significance. Data analysis was performed with GraphPad Prism. Alpha (α) was set at 0.05 for all statistical tests and data with a p ≤ 0.05 were considered statistically significant.

## Results

### Sun Exposure and CFH Expression in Normal Tissue

To assess the effect of sun exposure on CFH and CFI, we interrogated all available data in the GTEx portal. In the dataset of 473 exposed (lower leg) and 387 non-exposed (suprapubic) unpaired patient samples, CFH mRNA expression is higher in exposed skin than non-exposed skin (p < 0.0001) but no difference in CFI mRNA levels was seen.

**Figure 1:**
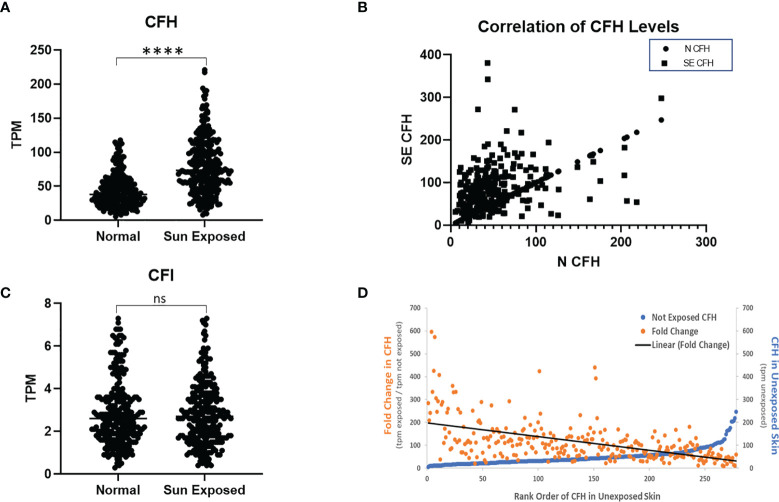
CFH Transcript Number is Increased in Sun-Exposed Skin. GTEx data of 278 paired sun exposed (lower leg) and non-sun exposed (suprapubic) patient samples were analysed. CFH levels are increased in exposed skin (p<0.0001, two-tailed Wilcoxon matched-pairs signed rank test) but not CFI **(A, C)**. Levels of CFH mRNA (TPM) in exposed skin correlates with unexposed skin (p<0.0001, Spearman’s rank-order correlation) **(B)**. When sorted by non-exposed CFH levels, the levels and fold increase in paired exposed samples decreases with the exception being at the highest values of CFH in non-exposed skin **(D)**. (****p<0.0001; ns, not significant).

To determine the effects of sun exposure using paired patient samples, we analysed the subset of 278 subjects with GTEx values for both sun-exposed and non-exposed skin. Analysis of these paired data suggests that CFH mRNA in exposed skin increased significantly over non-exposed skin (p < 0.0001) (**Figure 1A**). CFH mRNA in non-exposed skin correlates with levels in exposed skin (coefficient = 0.35, p < 0.0001) (**Figure 1B**) which suggests that complement levels before sun exposure significantly contribute to CFH levels after sun exposure. Levels of CFI mRNA were much lower than CFH mRNA and no difference in CFI mRNA was seen between exposed and non-exposed levels (**Figure 1C**).

To investigate these findings further, we next analysed the data from the 278 subjects with paired data by initial value of CFH mRNA in unexposed skin. While most initial CFH mRNA values are relatively low, those subjects with the highest values show a marked elevation in CFH mRNA in unexposed skin (blue line, **Figure 1D**). The mean fold change in CFH mRNA after sun exposure ranged from 9.2 to 596-fold with an average fold increase of 114.7 ± 5.4 tpm (transcripts per million ± SEM). These fold increase values gradually decrease with increasing CFH levels in unexposed skin (orange points and linear fit (black line), **Figure 1D**). Interestingly, while levels of CFI were not different between sun-exposed vs non-exposed skin, the correlative pattern was also seen in the CFI data and, suggestive of a link between these complement factors in paired patient samples, CFH levels correlate with CFI levels (coefficient = 0.58, p < 0.0001 in non-exposed and coefficient = 0.37, p < 0.0001 for exposed, data not shown).

### CFH in cSCC Samples

**Figure 2:**
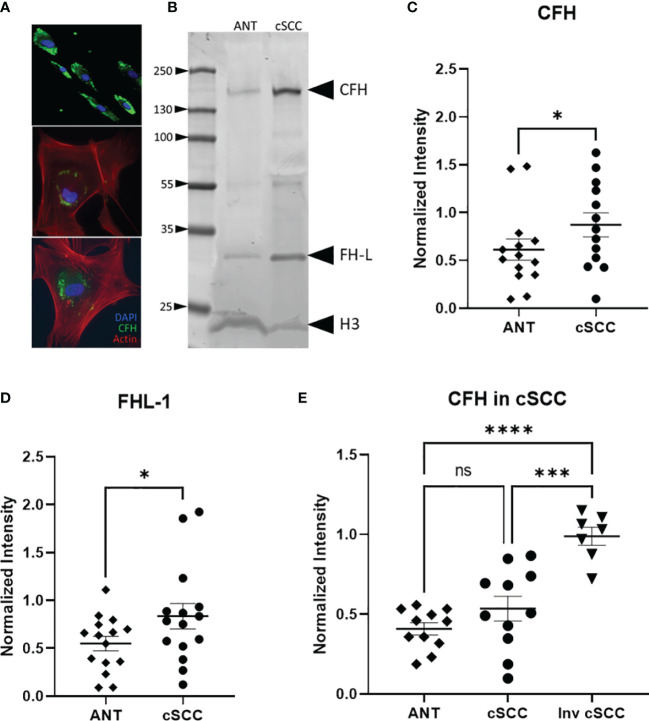
CFH and FH-L Expression in cSCC is Increased. Immunofluorescent microscopy of cells cultured from cSCC tissue show CFH staining (green) in cytosolic vesicles **(A)**. Bands at the expected molecular weight for CFH and Factor H-like (FH-L) are detected in both adjacent normal tissue and cSCC samples **(B)**. The ratio of CFH and FH-L band intensities, normalized to histone H3 intensity, was higher in cSCC tissue compared to ANT [**(C)**, p=0.031 & **(D)**, p=0.034, respectively]. In paired samples for non-invasive cSCC, CFH levels normalized to GAPDH are not significant. When CFH levels in ANT are compared to invasive cSCC, the difference is highly significant [**(E)**, p<0.0001]. (*p<0.05; ***p=0.0001; ****p<0.0001; ns, not significant).

Biopsy proven cSCC and adjacent normal tissue (ANT) from sun exposed skin were collected during routine Mohs micrographic surgery as previously described and primary cultures were established for both cSCC and ANT samples (21). Patients in the population had diagnoses of cSCC in situ, cSCC, early invasive cSCC, and invasive cSCC. As excised cSCC samples were typically small and had clean margins, it is expected that the cSCC samples analysed in this work contain a significant amount of ANT. This was confirmed by the observation that 75% of sections from excised tumor samples showed no evidence of cSCC after H&E staining as they sampled ANT removed with the biopsy proven cSCC (data not shown). We verified that CFH is produced by cells cultured from these tumor and adjacent normal tissue samples using immunofluorescent staining for CFH (**Figure 2A**). Cells in the mixed explant culture appear to synthesize CFH (top panel) and this staining appears to be in intracellular secretory vesicles (bottom panels). The intense punctate staining suggests that the majority of CFH may be contained within intracellular vesicles. In contrast, CFI was detected in positive control serum samples by immunoblotting but not reproducibly detected in cultured cells by immunofluorescence or in tissues samples by immunoblotting suggestive of lower expression levels of CFI in these samples consistent (data not shown). CFH was detected in immunoblots of cSCC and ANT tissue samples with patient serum included on the immunoblot as a positive control (**Figure 2B**). The differential splice product of the CFH gene, known as Factor H-like (FH-L), is also detected in our analysis. As FH-L retains key complement regulatory activities (7, 23), we included this product in the analysis.

Analysis of band intensities indicates a 1.76- and 1.53-fold increase in expression for CFH and FHL-1 respectively when compared to paired ANT (p = 0.031, n=13 and p = 0.034, n=15 respectively) (**Figures 2C, D**). Although the magnitude of the difference is small, as noted above, the analysed Mohs samples contained a significant amount of normal tissue which may act to decrease the magnitude of the change in CFH seen in the cSCC samples. Tissues included in these analyses were derived from 7 patients diagnosed with cSCC in situ, 4 invasive cSCC, and 2 early invasive cSCC samples. CFH expression in invasive cSCC tissues increases 1.17-fold over non-invasive cSCC (p = 0.0001, n=12 cSCC, n=7 invasive cSCC). When compared to unpaired ANT, levels of CFH are 2.10 fold higher in invasive cSCC despite the large amount of non-cSCC included in the Mohs samples (p < 0.0001, n=12 ANT, n=7 invasive cSCC) (**Figure 2E**).

### Immunomodulation in cSCC Samples

**Figure 3:**
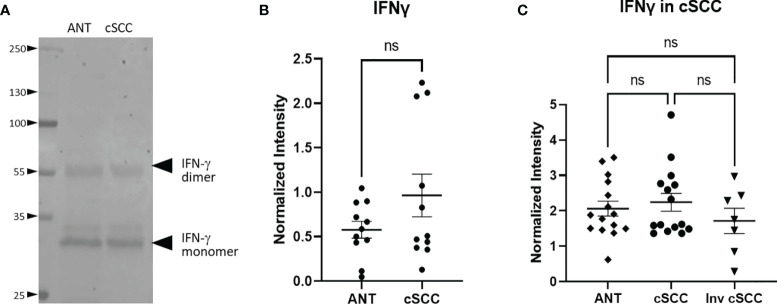
IFN-γ Expression in cSCC is Unchanged. Bands at the expected molecular weight for glycosylated IFN-γ monomer and IFN-γ dimer are detected at in both adjacent normal tissue and cSCC samples **(A)**. The ratio of intensity for both IFN-γ bands in paired samples was normalized to histone H3 intensity and is not significant (ns) **(B)**. The ratio of intensity for both IFN-γ bands in unpaired non-invasive and invasive cSCC samples normalized to GAPDH compared to control was also not significant (ns) **(C)**.

Levels of interferon gamma (IFN-γ) in the patient-derived cSCC samples above was quantitated next. This pro-inflammatory cytokine may mark an effective immune response and synthesis of CFH in keratinocytes has been reported to be under the control of IFN-γ (5–7). Immunoblotting revealed bands associated with the monomer and the biologically active glycosylated dimer form (**Figure 3A**, **Supplementary Figure 1**) (24, 25). After quantitation of both IFN-γ bands, no significant change in the level of IFN-γ between paired cSCC and ANT when normalized to histone H3 was detected (p = 0.150, n=11) (**Figure 3B**). In addition, when normalized to GAPDH, neither non-invasive or invasive cSCC IFN-γ levels change (p = 0.8511 and p = 0.687 respectively, n=15 ANT and noninvasive, n=7 invasive) (**Figure 3C**).

**Figure 4:**
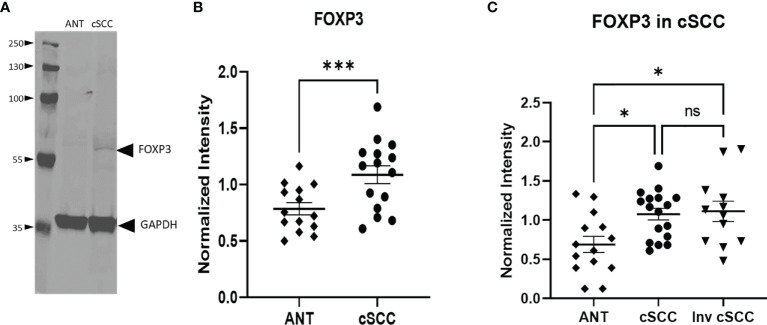
Levels of FOXP3 are Increased in cSCC. Immunoblots detected a band at the expected molecular weight for FOXP3 in both adjacent normal tissue and cSCC samples **(A)**. The ratio of FOXP3 band intensity normalized to GAPDH intensity was higher in cSCC tissue compared to paired ANT [**(B)**, p<0.001] and non-invasive cSCC compared to unpaired ANT but there is no difference between non-invasive and invasive cSCC **(C)**. (*p<0.05; ***p=0.0001; ns, not significant).

The transcription factor FOXP3 is often used to detect the presence of regulatory T cells which are reflective of a dampened immune response. Immunoblotting revealed a band at the expected molecular weight for FOXP3 (**Figure 4A**, **Supplementary Figure 2**). Levels of FOXP3 were quantitated in both non-invasive and invasive cSCC and compared to levels in adjacent normal tissue in our clinic samples. As seen in **Figure 4B**, paired non-invasive cSCC shows a significant increase in this transcription factor (p<0.001). However, when FOXP3 levels in invasive cSCC are compared to non-invasive, there is no change (**Figure 4C**). These data are consistent with an increase in FOXP3 levels during the initial stages of tumor development but FOXP3 may not be playing a role in promoting tumor progression once cancer has developed.

### Relative CFH and FOXP3 in cSCC Samples by IHC

We next compared levels of CFH and FOXP3 in our patient-derived ANT and cSCC samples to commercially available arrays of advanced cSCC by immunohistochemistry (IHC). This allows us to directly assess levels of these proteins in newly diagnosed cSCC excised in a dermatology clinic as part of routine practice (ALBR Samples), which may not be as progressed, with the more advanced cSCC samples utilized in most studies. In addition, as the samples in these analyses retain the tissue integrity, they allowed for determination of colocalization of these proteins within the tumor tissues.

**Figure 5:**
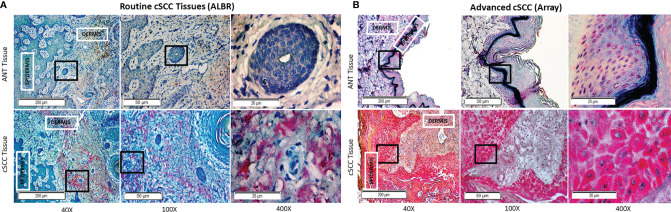
CFH in Routine Mohs and Advanced cSCC Samples by IHC. cSCC removed from routine clinic patients by Mohs surgery [fixed after cryosectioning; **(A)**] and an array of advanced cSCC [formalin fixed; **(B)**] were labeled with mouse anti-CFH (OX-24) and an AP-conjugated secondary antibody with permanent red stain. Mayer’s Hematoxylin was used as a counterstain (nuclei). The degree of CFH staining in the ANT and cSCC samples was semi-quantitatively determined by using a 0-3+ scale, with 0 indicating no staining, 1+ indicating <10% staining, 2+ indicating 10-50% staining, and 3+ indicating >50% staining. ANT samples [**(A, B)**, top panels] were scored as either 0 or 1+, the Mohs cSCC samples [**(A)**, bottom panel] were scored as 2+, and the advanced cSCC samples [**(B)**, bottom panel] were scored as 3+. The boxes within the 40x and 100x images delineate the tissue location shown in the 100x and 400x images, respectively.

As shown in **Figure 5**, levels of CFH seen in ANT were compared to cSCC in routine clinic (ALBR; **Figure 5A**) and advanced cSCC (Array; **Figure 5B**) samples by IHC. As CFH is a secreted protein which may be detected intracellularly as well as bound to the extracellular matrix, no specific localization was expected. Consistent with being secreted by keratinocytes, higher levels of CFH appear to be localized in epidermal than dermal layers (red color) in both the ANT and cSCC samples. Suggestive of a relationship to sun exposure, CFH appears higher (1+) in sun damaged regions, as easily seen in the ALBR ANT sample but also in the Array ANT sample. Comparing cSCC tissues, CFH staining appears more intense than in ANT tissues, increasing to 2+ in routine clinic samples (ALBR cSCC) and to 3+ in advanced samples (Array cSCC). Additional IHC images at 400x magnification are provided in **Supplementary Figures 3**, **4**. Thus, despite the limitation of variations in color due to the different sources and initial preparation of slides, these data suggest that more advanced tumors have higher levels of CFH than our patient-derived samples used in this study.

**Figure 6:**
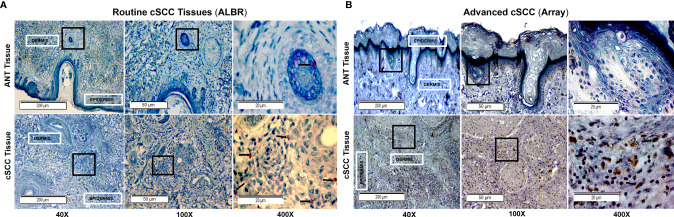
FOXP3 in Routine Mohs and Advanced cSCC Samples by IHC. cSCC removed from routine clinic patients by Mohs surgery [fixed after cryosectioning; **(A)**] and an array of advanced cSCC [formalin fixed; **(B)**] were labeled with a rabbit monoclonal anti-FOXP3 (Cell Marque) and stained with an HRP-conjugated secondary antibody and DAB stain (for Array samples) and with an AP-conjugated secondary antibody and permanent red stain (for ALBR samples). Mayer’s Hematoxylin was used as a counterstain (nuclei). The degree of FOXP3 staining in the ANT and cSCC samples was semi-quantitatively determined by using a 0-3+ scale, with 0 indicating no staining, 1+ indicating <10% staining, and 2+ indicating 10-50% staining, and 3+ indicating >50% staining. ANT samples [**(A, B)**, top panels] were scored as 0, the Mohs cSCC samples [**(A)**, bottom panel; arrows in the 400x image denote positive nuclear localization] were scored as 1+, and the advanced cSCC samples [**(B)**, bottom panel; arrows in the 400x image denote positive nuclear localization] were scored as 2+. The boxes within the 40x and 100x images delineate the tissue location shown in the 100x and 400x images, respectively.

Next, levels of FOXP3 in ANT and cSCC tissues from routine clinic (ALBR; **Figure 6A**) and advanced cSCC samples (Array; **Figure 6B**) were determined using IHC. As a transcription factor associated with development of regulatory T cells and, due to our results showing elevated CFH in these tumor samples, we expected FOXP3 staining to be more intense within the immune infiltrate surrounding tumor tissue (arrows). As shown in **Figure 6B**, moderate FOXP3 staining (2+) is seen in more advanced cSCC (Array samples). Additional IHC images at 400x magnification are provided in **Supplementary Figures 5**, **6**. Although there are similar limitations of color variation due to different initial slide preparation as in **Figure 5**, there appears to be substantially less FOXP3 staining in routine clinic samples (**Figure 6A**) although we do see more FOXP3 staining in our less advanced patient-derived cSCC tissues (1+) than in ANT tissue (0). Our patient-derived cSCC tissues may not be as advanced as the tissues used for the commercial Array slides, which may explain the lack of significance between the two patient-derived cSCC tissues (**Figure 4C**) while the more advanced cSCC images show abundant FOXP3 staining using IHC (**Figure 6B**). Results suggest the FOXP3 positive immune infiltrate is increased in the advanced cSCC samples when compared to those in routine clinic samples, again correlated to the increased CFH observed in these advanced cSCC samples.

## Discussion

Data presented here strengthen the link between cSCC and CFH. By focusing on cSCC tumors excised from patients seen in routine Mohs microsurgery patients in an Arizona-based dermatology practice (where the typical patient likely received significant sun exposure), the link between CFH is extended to newly diagnosed and non-invasive cSCC. Although the observed CFH elevation was small, this is perhaps due to the relatively small size of the tumor and the amount of normal tissue included in the patient samples excised in the Mohs procedure. Indeed, we suspect that this may be the reason that CFI was not reliably detected in our samples as others have found that CFI levels in tissues are lower than CFH and consistent with GTEx data (**Figure 1**). Our GTEx analysis showed a significant difference in CFH levels between sun-exposed vs non-exposed tissues, suggesting that sun exposure influences CFH levels. While we observed increases in CFH levels in our patient-derived tissues consistent with sun exposure, we showed that our cSCC tissues express higher levels of CFH than ANT tissues. However, as our samples were paired, with each pair receiving the same level of sun exposure, the elevation in CFH in cSCC compared to ANT cannot be explained by sun exposure alone. Consistent with a role in progression, more advanced cSCC show markedly more dramatic increases in CFH and FOXP3 by IHC than the routine patient-derived samples. Thus, these data suggest that elevation in CFH appears early in the development of cSCC and is significant despite these complicating factors. Comparison of these data to an invasive cSCC set suggests a link with cSCC progression and raises the possibility that CFH levels may be an important prognostic factor in assessing cSCC.

As our collective data sets provide support for sun exposure affecting overall levels of CFH, we suggest that immune modulation is an early event in the development of cSCC. This finding is not unexpected as an increase in CFH is expected to reduce both innate and adaptive immune responses to tumors, a necessary step in tumor progression. Furthermore, the fact that IFN-γ levels do not increase in our cSCC samples when compared to ANT may be consistent with an ineffective immune response. It is interesting that we do not see elevated levels of IFN-γ as this cytokine has been shown to increase CFH secretion (5, 7). It is possible that in the early stages of an immune response to developing tumors, IFN-γ secretion leads to increased CFH expression which ultimately derails the immune response and allows tumor progression. Alternatively, elevated CFH may be downstream of sun exposure rather than increased IFN-γ levels. That said, it is also plausible that our immunoblotting techniques were not sensitive enough to detect any increase in IFN-γ (particularly as our cSCC samples contain substantial amounts of ANT). However, we suggest that the putative increase in regulatory T cells is more consistent with insufficient IFN-γ levels for an effective immune response. Specifically, although the pro-inflammatory tumor infiltrate is not directly assessed, we do detect increased levels of FOXP3 within cSCC samples (**Figure 6**). These data are consistent with published results (26). Although FOXP3 is often a marker for regulatory T cells, other cell types have been reported to transiently express FOXP3, including regulatory B cells and M2 macrophages [as more recently reviewed in (27)], which have been shown to be elevated in various tumors and are associated with anti-inflammatory and immunosuppressive roles (28–31). While we cannot definitively confirm all the FOXP3+ cells are regulatory T cells, we can conclude that the environment within the cSCC tissues is immunosuppressive compared to ANT and may be indicative of a reduced immune response that would favor tumor growth, regardless of cell lineage. Given that sun exposure may lead to CFH secretion by a mechanism which may or may not be linked to IFN-γ, it is impossible to determine given the nature of the tissues samples generated by Mohs surgery without altering the standard of care of these patients.

This work documents both an increase in CFH and FOXP3 in cSCC but does not directly address the relationship between these two findings. Published work suggests a plausible causal link between increased CFH secreted from cSCC and immunoevasion as suggested by increased FOXP3 levels. Expression of CFH by keratinocytes and cSCC cells lines has been well documented (5, 7) and it is expected that the increased expression of this complement regulatory protein would reduce levels of anaphylatoxins within the tumor, shifting the immune response from an effective Th1-mediated to an ineffective regulatory T cell response. However, how this altered CFH might affect the balance between effective and ineffective immune responses is not clear.

Although not immune cells, growing evidence suggests that cancer cells express anaphylatoxin receptors and are able to respond to increased anaphylatoxin levels. Specifically, a wide variety of cancers and cancer cell lines express C3aR and C5aR and respond by increased motility and activation of the ERK1/2 pathway to promote growth (32–37). Most relevant to this work, cultured cSCC respond to both CFH and CFI (5, 6) and the receptor for the more potent C5a can be detected in skin tissue and is expressed in skin cancer lines (11). Indeed, levels of C3aR and C5aR2 mRNA in the GTEx dataset increase with sun exposure while those of C5aR1 do not (**Supplementary Figure 7**). However, particularly as mRNA levels are very low, expression of these receptors must be verified with validated antibodies. Given that increased CFH and CFI would decrease C3a and C5a levels, increased CFH would not favor tumor progression through canonical complement pathways.

Data presented here helps to solidify the relationship between CFH and tumorigenesis but they also raise many questions about the role of CFH in cancer progression. Specifically, in addition to its role in cSCC, a role for CFH has been described for hepatocellular and clear cell renal cell carcinomas (ccRCC) but not does not appear to promote squamous cell lung carcinoma (6, 38). In addition, CFH may promote ccRCC but does affect tubular cells from which ccRCC arise (6, 39). Thus, the roles for complement proteins are complex and it is difficult to predict how alterations in CFH will ultimately affect tumorigenesis. Indeed, recent data expanding on the link between a CFH allele and the risk for age related macular degeneration has revealed distinct intracellular roles for CFH in metabolism and response to oxidative stress and CFH knock-down may alter NFkB and p53 function (18, 20). Although the complex role of CFH in cancer cells underscores the importance of further investigation of complement in the immune surveillance of cancers. However, the current samples do not allow us to clearly distinguish between intracellular and extracellular roles of CFH and additional studies with different experimental approaches are warranted.

There are many remaining questions regarding the role of CFH in cSCC and clarification of these points may have direct impact on treatment of patients with cSCC. To clarify whether the increase in CFH contributes to or is a result of tumor development, it will be important to establish signaling through anaphylatoxin receptors in cSCC and solidify the evidence of immune modulation. The ability of IFN-γ to increase CFH secretion by keratinocytes documented in cell lines can be replicated in patient samples needs to be investigated. This latter point is of particular importance as a current therapy, imiquimod (used in the treatment actinic keratosis and some cSCC), is associated with enhancing IFN-γ production to mount an effective immune response (40) through altering effector T cell responses (41). However, it should be noted that IFN-γ plays a complex role in immunity in that it both activates effector T cells as well as potentially being involved in induced regulatory T cells (42).

## Conclusions

CFH may be elevated in cSCC tumors excised from patients seen in a routine Mohs microsurgery. This elevation in CFH appears to be independent of sun exposure and may act through derailing an effective immune response. Immune checkpoint therapies targeting anaphylatoxin receptors may be an effective treatment for cSCC in the future.

## Data Availability Statement

The datasets presented in this study can be found in online repositories. The names of the repository/repositories and accession number(s) can be found below: The Genotype-Tissue Expression (GTEx) Project portal at https://gtexportal.org/home/.

## Ethics Statement

The studies involving human participants were reviewed and approved by Western IRB (WIRB Protocol #20142461) to Affiliated Laboratories BioRepository. The patients/participants provided their written informed consent to participate in this study. Approval was also obtained from Midwestern University IRB (AZ#807).

## Author Contributions

EJ, CU, and AP conducted experiments and contributed to data analysis. SE, RA, KL, and EH analysed data and edited the manuscript. KL and EH conceived of the project and coordinated the research endeavours. All authors have approved the manuscript.

## Funding

This research was funded by MWU Research Facilitation Grants to KL and EH and by graduate student funds to EJ.

## Conflict of Interest

The authors declare that the research was conducted in the absence of any commercial or financial relationships that could be construed as a potential conflict of interest.

## Publisher’s Note

All claims expressed in this article are solely those of the authors and do not necessarily represent those of their affiliated organizations, or those of the publisher, the editors and the reviewers. Any product that may be evaluated in this article, or claim that may be made by its manufacturer, is not guaranteed or endorsed by the publisher.

## Supplementary Material

Supplementary Figure 1 IFN-γ With Loading Controls GAPDH and Histone H3. Immunoblot of ANT and cSCC samples probed with anti-IFN-γ, anti-GAPDH, and anti-H3. Bands corresponding to dimer and monomer IFN-γ, and both loading controls, were observed.

Supplementary Figure 2 FOXP3 and Loading control GAPDH. Immunoblot of ANT and cSCC samples probed with anti-FOXP3 and anti-GAPDH. A longer exposure time was needed to visualize FOXP3 than GAPDH.

Supplementary Figure 3 CFH in Routine Mohs cSCC Samples by IHC. Additional images at 400x magnification of cSCC removed from routine clinic patients by Mohs surgery (fixed after cryosectioning). Sections were labeled with mouse anti-CFH (OX-24) and an AP-conjugated secondary antibody with permanent red stain. Mayer’s Hematoxylin was used as a counterstain (nuclei).

Supplementary Figure 4 CFH in Advanced cSCC Samples by IHC. Additional images at 400x magnification of advanced cSCC (formalin fixed) array slides. Sections were labeled with mouse anti-CFH (OX-24) and an AP-conjugated secondary antibody with permanent red stain. Mayer’s Hematoxylin was used as a counterstain (nuclei).

Supplementary Figure 5 FOXP3 in Routine Mohs cSCC Samples by IHC. Additional images at 400x magnification of cSCC removed from routine clinic patients by Mohs surgery (fixed after cryosectioning). Sections were labeled with were labeled with a rabbit monoclonal anti-FOXP3 (Cell Marque) and stained with an AP-conjugated secondary antibody and permanent red stain. Mayer’s Hematoxylin was used as a counterstain (nuclei).

Supplementary Figure 6 FOXP3 in Advanced cSCC Samples by IHC. Additional images at 400x magnification of advanced cSCC (formalin fixed) array slides. Sections were labeled with a rabbit monoclonal anti-FOXP3 (Cell Marque) and stained with an HRP-conjugated secondary antibody and DAB stain. Mayer’s Hematoxylin was used as a counterstain (nuclei).

Supplementary Figure 7 Expression of Complement Receptors in GTEx Datasets. Analysis of unpaired GTEx data from sun exposed vs non-sun exposed shows that sun exposed tissue has a significant increase in mRNA expression of C3aR (p=0.0014) and C5aR2 (p=0.0005) compared to non-sun exposed skin. mRNA expression of C5aR1 was not significantly different in this analysis (p=0.4726).
