# Supplementary figures and images for: Complement Factor H in cSCC: Evidence of a Link Between Sun Exposure and Immunosuppression in Skin Cancer Progression
Source: Front Oncol. 2022 Feb 10;12:819580. doi: 10.3389/fonc.2022.819580 (PMC8869607; doi:10.3389/fonc.2022.819580)

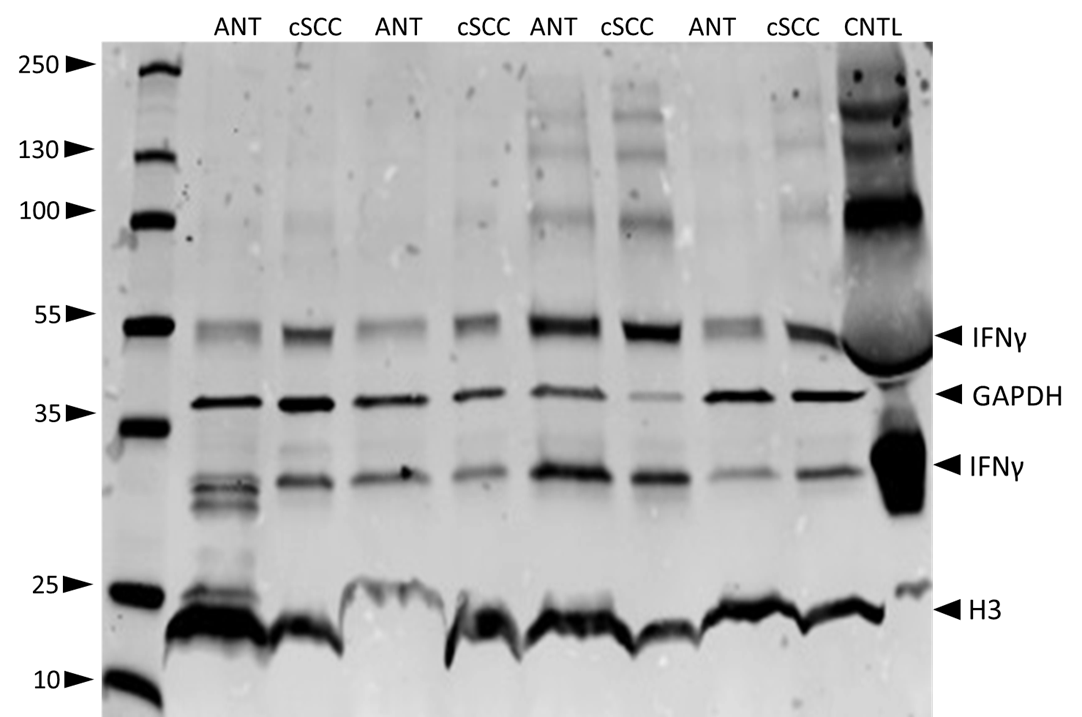

Supplement: Supplementary Figure 1 — IFN-γ With Loading Controls GAPDH and Histone H3. Immunoblot of ANT and cSCC samples probed with anti-IFN-γ, anti-GAPDH, and anti-H3. Bands corresponding to dimer and monomer IFN-γ, and both loading controls, were observed. [file Image_1.tif]

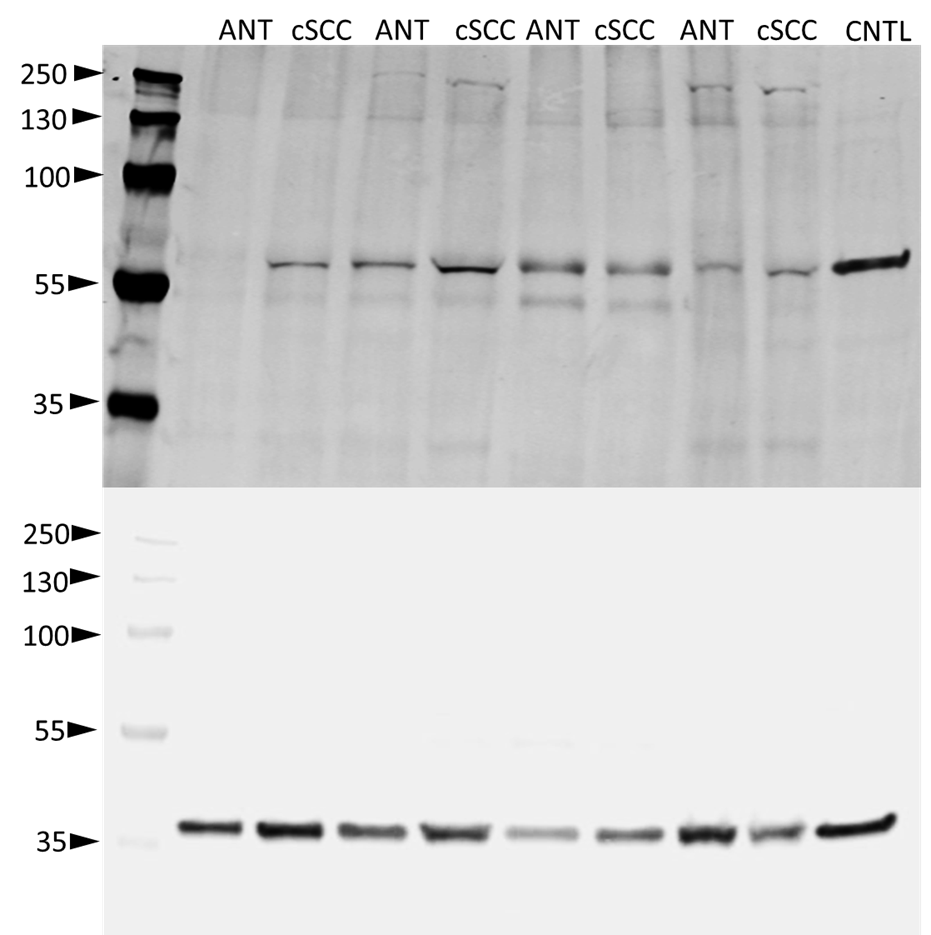

Supplement: Supplementary Figure 2 — FOXP3 and Loading control GAPDH. Immunoblot of ANT and cSCC samples probed with anti-FOXP3 and anti-GAPDH. A longer exposure time was needed to visualize FOXP3 than GAPDH. [file Image_2.tif]

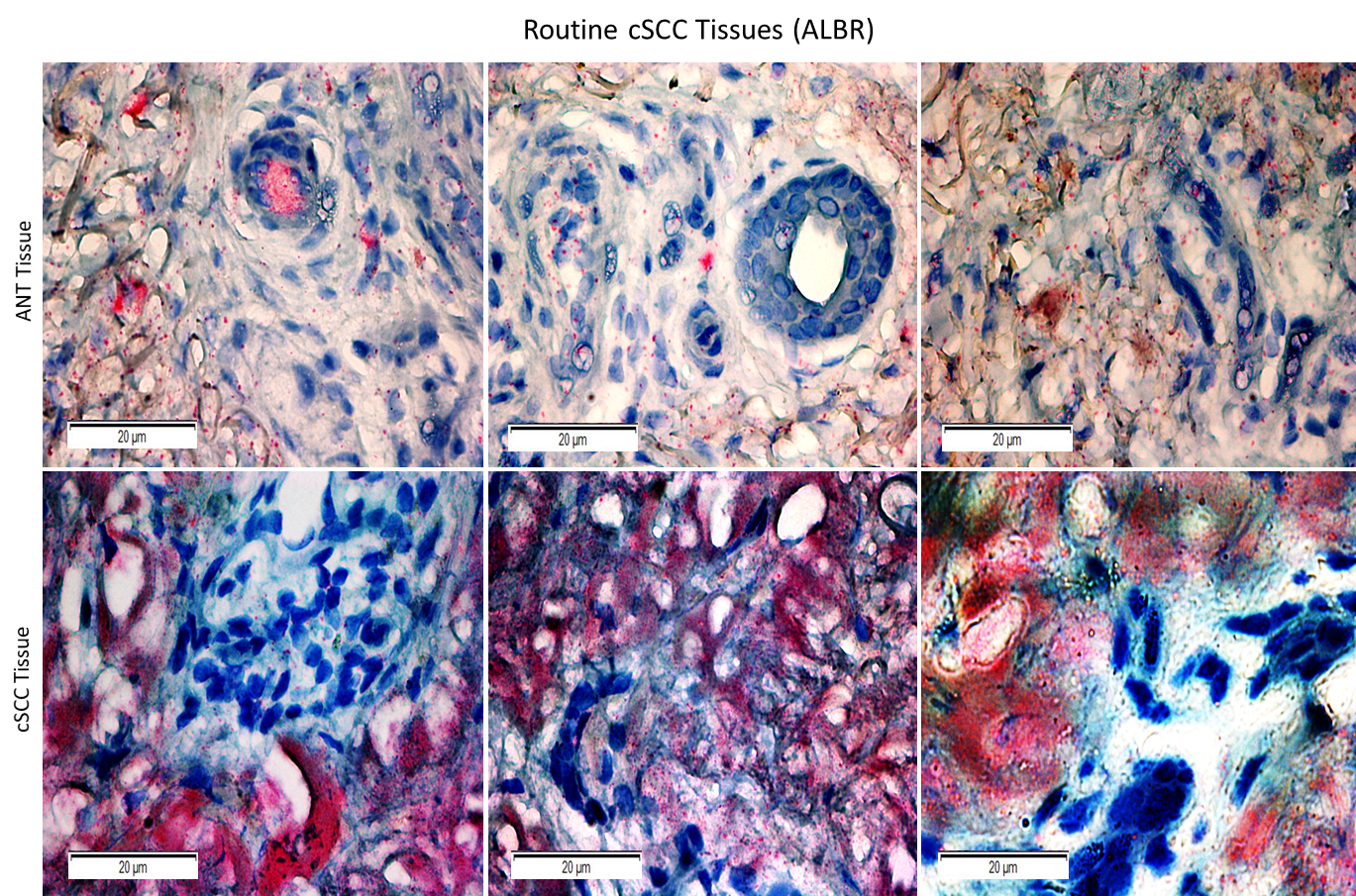

Supplement: Supplementary Figure 3 — CFH in Routine Mohs cSCC Samples by IHC. Additional images at 400x magnification of cSCC removed from routine clinic patients by Mohs surgery (fixed after cryosectioning). Sections were labeled with mouse anti-CFH (OX-24) and an AP-conjugated secondary antibody with permanent red stain. Mayer’s Hematoxylin was used as a counterstain (nuclei). [file Image_3.tif]

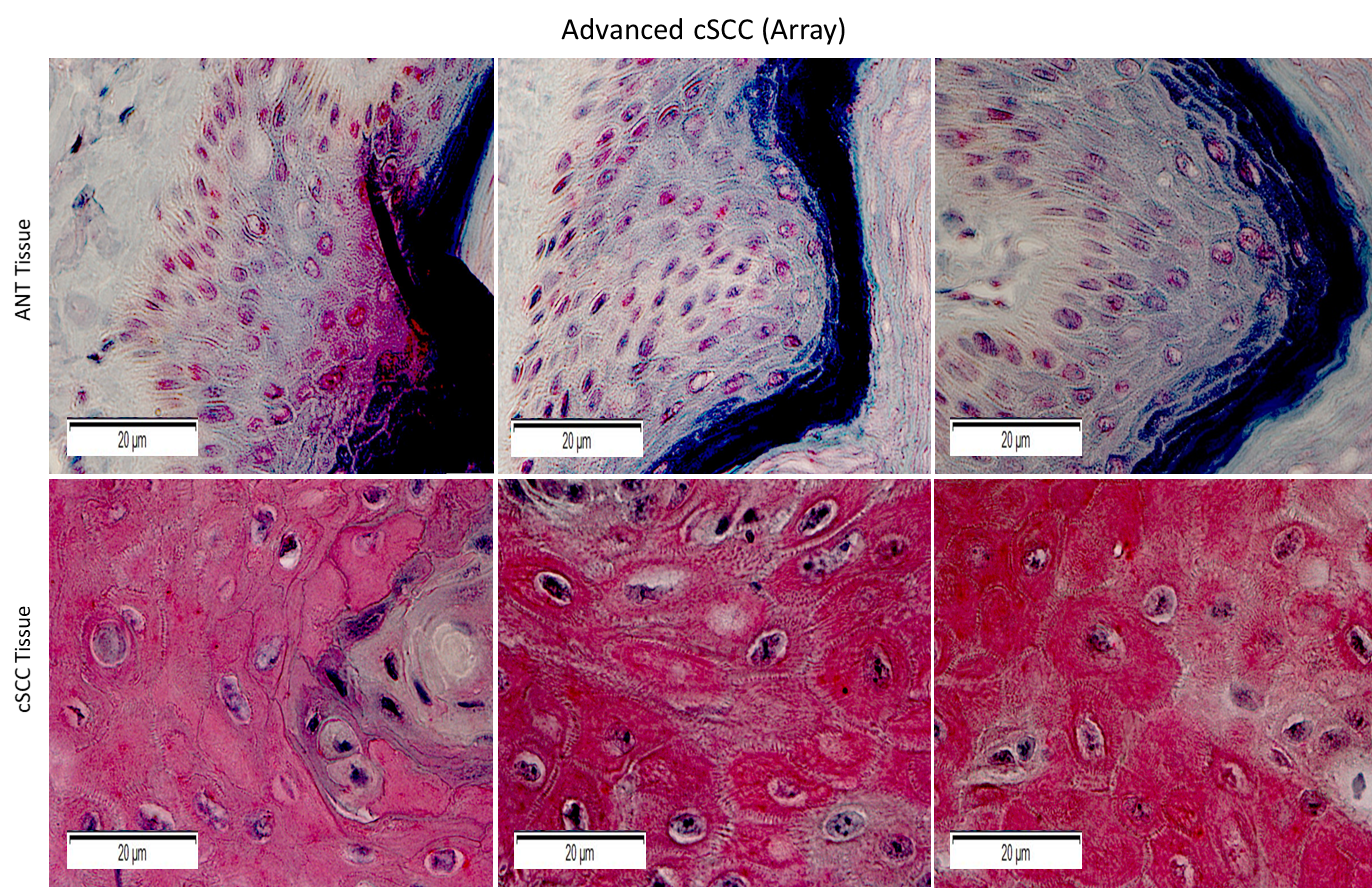

Supplement: Supplementary Figure 4 — CFH in Advanced cSCC Samples by IHC. Additional images at 400x magnification of advanced cSCC (formalin fixed) array slides. Sections were labeled with mouse anti-CFH (OX-24) and an AP-conjugated secondary antibody with permanent red stain. Mayer’s Hematoxylin was used as a counterstain (nuclei). [file Image_4.tif]

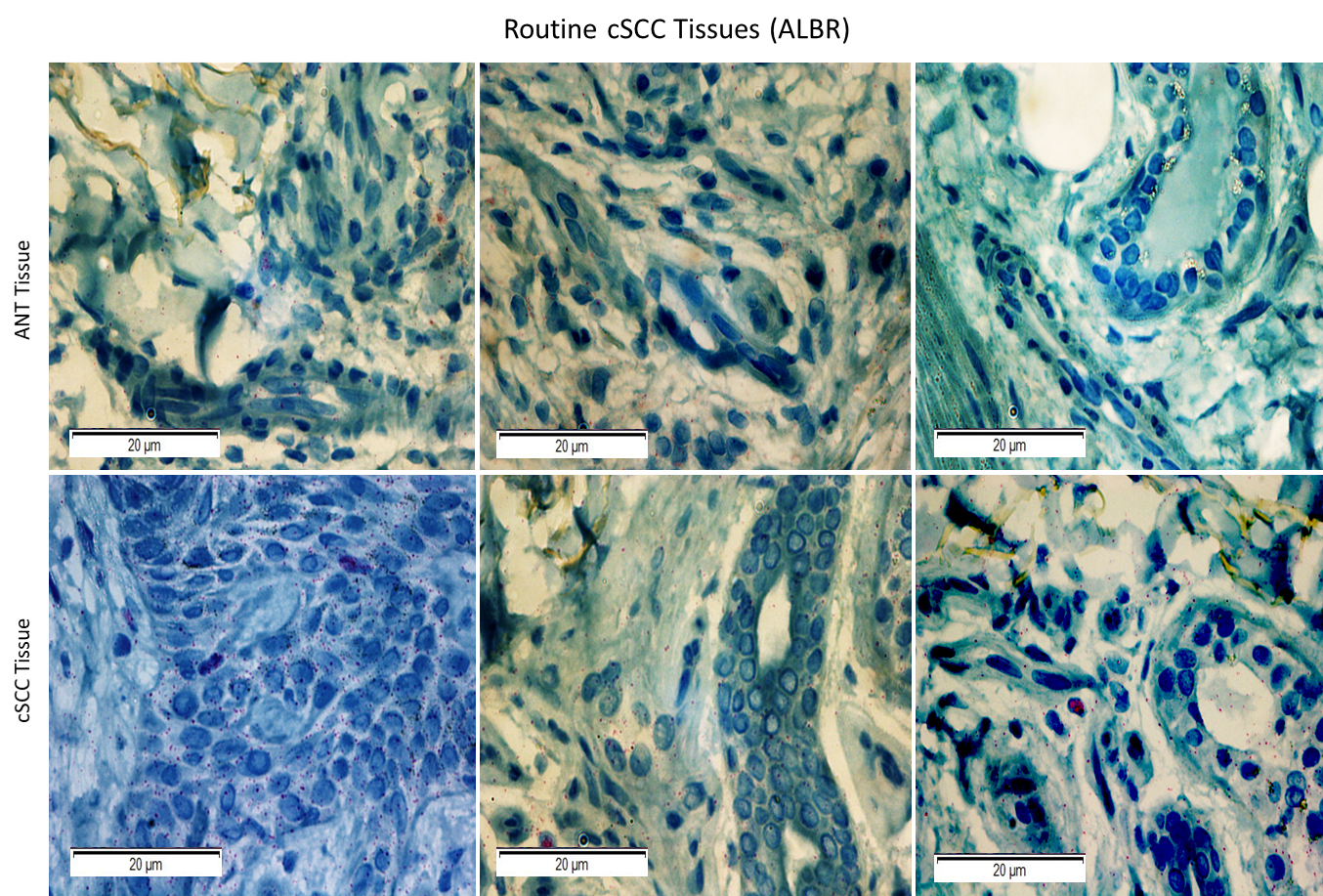

Supplement: Supplementary Figure 5 — FOXP3 in Routine Mohs cSCC Samples by IHC. Additional images at 400x magnification of cSCC removed from routine clinic patients by Mohs surgery (fixed after cryosectioning). Sections were labeled with were labeled with a rabbit monoclonal anti-FOXP3 (Cell Marque) and stained with an AP-conjugated secondary antibody and permanent red stain. Mayer’s Hematoxylin was used as a counterstain (nuclei). [file Image_5.tif]

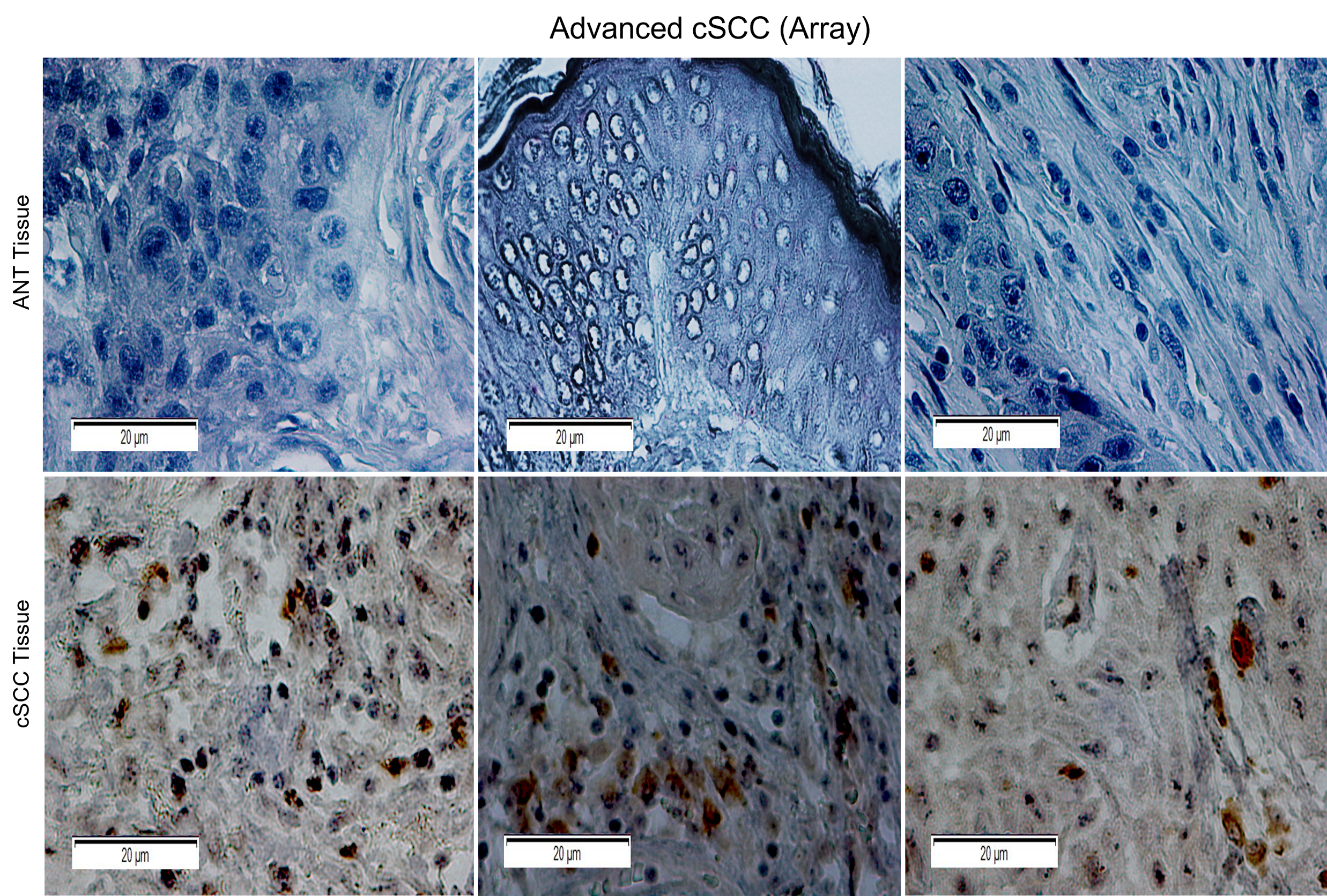

Supplement: Supplementary Figure 6 — FOXP3 in Advanced cSCC Samples by IHC. Additional images at 400x magnification of advanced cSCC (formalin fixed) array slides. Sections were labeled with a rabbit monoclonal anti-FOXP3 (Cell Marque) and stained with an HRP-conjugated secondary antibody and DAB stain. Mayer’s Hematoxylin was used as a counterstain (nuclei). [file Image_6.tif]

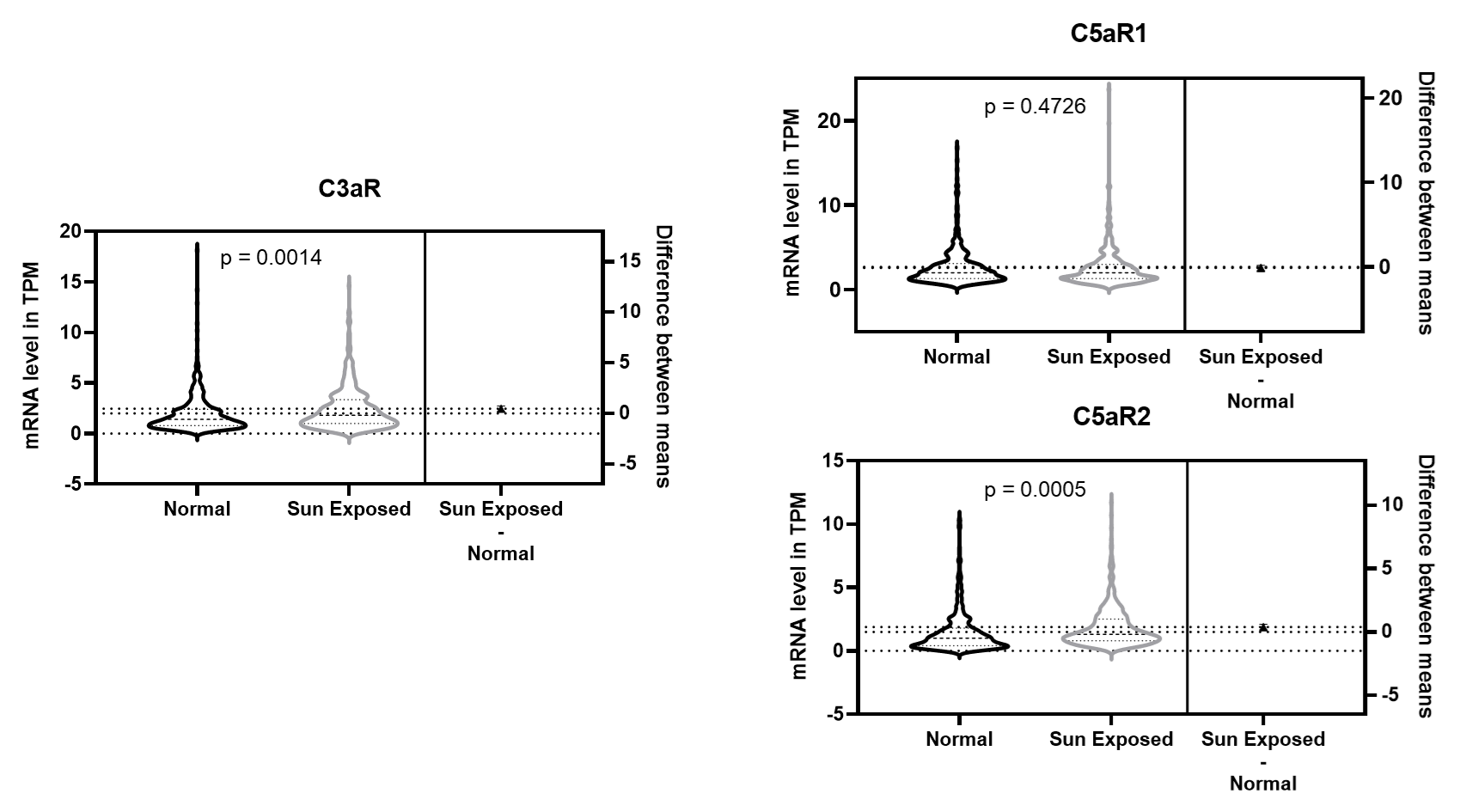

Supplement: Supplementary Figure 7 — Expression of Complement Receptors in GTEx Datasets. Analysis of unpaired GTEx data from sun exposed vs non-sun exposed shows that sun exposed tissue has a significant increase in mRNA expression of C3aR (p=0.0014) and C5aR2 (p=0.0005) compared to non-sun exposed skin. mRNA expression of C5aR1 was not significantly different in this analysis (p=0.4726). [file Image_7.tif]
